# Expression of resistance gene analogs in woodland strawberry (*Fragaria vesca*) during infection with *Phytophthora cactorum*

**DOI:** 10.1007/s00438-016-1232-x

**Published:** 2016-07-22

**Authors:** Xiao-Ren Chen, May Bente Brurberg, Abdelhameed Elameen, Sonja Sletner Klemsdal, Inger Martinussen

**Affiliations:** 1 Norwegian Institute of Bioeconomy Research, Box 115, 1431 Ås, Norway; 2 College of Horticulture and Plant Protection, Yangzhou University, Wenhui Eastern Road 48, Yangzhou, 225009 Jiangsu Province China

**Keywords:** Strawberry, Crown rot disease, *Phytophthora cactorum*, NBS-LRR genes, Resistance gene analogs

## Abstract

Important losses in strawberry production are often caused by the oomycete *Phytophthora cactorum*, the causal agent of crown rot. However, very limited studies at molecular levels exist of the mechanisms related to strawberry resistance against this pathogen. To begin to rectify this situation, a PCR-based approach (NBS profiling) was used to isolate strawberry resistance gene analogs (RGAs) with altered expression in response to *P. cactorum* during a time course (2, 4, 6, 24, 48, 96 and 192 h post-infection). Twenty-three distinct RGA fragments of the NB-LRR type were identified from a resistance genotype (Bukammen) of the wild species *Fragaria vesca*. The gene transcriptional profiles after infection showed that the response of most RGAs was quicker and stronger in the resistance genotype (Bukammen) than in the susceptible one (FDP821) during the early infection stage. The transcriptional patterns of one RGA (RGA109) were further monitored and compared during the *P. cactorum* infection of two pairs of resistant and susceptible genotype combinations (Bukammen/FDP821 and FDR1218/1603). The 5′ end sequence was cloned, and its putative protein was characteristic of NBS-LRR R protein. Our results yielded a first insight into the strawberry RGAs responding to *P. cactorum* infection at molecular level.

## Introduction

Plants are often attacked by a great number of pests, including viruses, bacteria, nematodes, insects, fungi, and oomycetes. However, they have evolved elaborate mechanism to protect themselves against the attackers (Dangl and Jones 2001). One of the efficient defense mechanisms is a type of immunity that is described by the gene-for-gene theory, which was proposed by Harold Henry Flor in the last century (1942, 1971). The induction of the plant defense response is considered to be initiated through the recognition of specific effectors encoded by *Avr* (avirulence) genes of the pathogens by the complementary products of the genes conferring resistance, the *R* genes of the host plants (Flor 1942, 1971). To date, more than 70 different genes for resistance (*R* genes) to major plant pathogens have been isolated and characterized from different plant species (Sharma et al. 2009; Gururani et al. 2012). Most of the *R* genes discovered in plants have conserved functional domains and can be assigned to one of the five major classes of *R* genes (Dangl and Jones 2001). The nucleotide-binding site (NBS)/leucine-rich repeats (LRRs) class is by far the predominant class, and their resistance gene analogs (RGAs) are numerous in plants and distributed over all chromosomes in some plant species (Meyers et al. 2002, 2003; Monosi et al. 2004).

The conserved primary structure of resistance gene sequences has often been used to isolate RGAs by PCR, with degenerate primers designed from the highly conserved motifs of these proteins (Sharma et al. 2009). The NBS profiling technique is an example of a motif-directed profiling technique that targets *R* genes and RGAs using degenerate primers that are homologous to the conserved motif sequences (P-loop, the kinase-2 motif, and the GLPL motif) in the NBS domain of the NBS-LRR class of *R* genes. The degenerate primers are designed to recognize specifically a large number of NBS-LRR gene family members (Van der Linden et al. 2004). This technique has been successfully used to identify *R* genes and RGAs in apple, lettuce, potato, tomato, wheat, and barley (Van der Linden et al. 2004; Calenge et al. 2005; Mantovani et al. 2006; Syed et al. 2006; Malosetti et al. 2007). In addition to being a powerful tool for the identification of *R*-genes, NBS profiling technique has been used to generate candidate gene markers linked to *R*-gene loci which can be used in marker-assisted breeding (Brugmans et al. 2008; Jacobs et al. 2010; Jo et al. 2011).

Strawberry is an important horticultural crop grown in many tropical, subtropical, and temperate areas throughout the world with an annual production of approximately 4.5 million tons (2012—FAOSTAT Agriculture Data, http://faostat.fao.org). The main cultivated species around the world is the octoploid *Fragaria* × *ananassa* Duch. (2*n* = 8*x* = 56) (Davis et al. 2007), while the most common native species, *F. vesca* L., is a diploid with 14 chromosomes (Oosumi et al. 2006). The annual strawberry production is strongly affected by phytopathogens, forcing the excessive use of chemicals to control them (Maas 1998). The destructive oomycete *Phytophthora cactorum* (Lebert and Cohn) Schröeter (1886) is a root pathogenic oomycete which causes crown rot in strawberry plants throughout the world (Erwin and Ribeiro 1996; Maas 1998). Crown rot disease has been an important limiting factor to successful strawberry production worldwide, because most cultivated strawberry cultivars are highly susceptible to attack by *P. cactorum* (Maas 1998; Eikemo et al. 2010; Chen et al. 2011). However, genotypes resistance to crown rot do exist (Eikemo et al. 2010), and management through host resistance has been considered as one of the best options available for crop protection.

Martínez-Zamora et al. (2004) reported for the first time on RGAs of the NBS-LRR class in strawberry. Seven distinct families of RGAs of the NBS-LRR type were identified from wild species *F. vesca* and *F. chiloensis*, and six different *F.* × *ananassa* cultivars, by genomic DNA amplification using degenerate primers. Jung et al. (2010) reported a cluster of four RGAs, contained in a strawberry (*F. vesca*) fosmid (34E24), with NBS and LRR domains, and conserved in all the five rosid genomes with which they compared them. More recently, a number of strawberry RGAs with NBS-LRR domain were predicted (Perazzolli et al. 2014; Zhong et al. 2015). At least 144 NBS-LRR genes reportedly exist in strawberry genome based on a systematic genome-wide survey (Zhong et al. 2015). These detected RGAs could in principle be involved in strawberry resistance. However, no experimental evidence exists to link the presence or absence of any particular *R* gene or RGA to disease resistance in strawberry.

Van de Weg (1997) found support for one single segregating dominant strawberry resistance gene (*Rpf2*), against the oomycete *Phytophthora fragariae,* while a dominant, two-gene model has been suggested in red raspberry (*Rubus ideaus*) resistance toward *Phytophthora rubi* (Pattison et al. 2007). For resistance to *P. cactorum* in stawberry, there are several reports indicating a more complex gene model (Shaw et al. 2006, 2008; Denoyes-Rothan et al. 2004). Focusing on a simpler system than the octoploid strawberry, e.g., a diploid model system, thus appears attractive to get an understanding of the nature and inheritance of the *Phytophthora* crown rot resistance.

The diploid woodland *F. Vesca* has been considered a model plant system for the *Rosaceae* family because of its attractive features, such as a small genome size (~240 Mb), available transformation protocol, released genome sequence, and short generation time (Alsheikh et al. 2002; Folta and Davis 2006; Shulaev et al. 2011). Genetic maps exist for both the diploid and octoploid strawberry (Davis and Yu 1997; Lerceteau-Köhler et al. 2003; Sargent et al. 2004, 2006; Cipriani et al. 2006; Folta and Davis 2006; Weebadde et al. 2008). A high degree of macrosynteny and colinearity between diploid and octoploid strawberry exists, and no major chromosomal rearrangements seem to have occurred (Folta and Davis 2006; Rousseau-Gueutin et al. 2008, 2009). It is thus reasonable to assume that these two strawberry species with their common ancestor employ similar resistance mechanisms against pathogen attacks, and it was recently found that diploid strawberry encompass a wide range of resistant phenotypes toward *P. cactorum* (Eikemo et al. 2010). Hence, in this study, the diploid strawberry was utilized to analyze strawberry resistance to *P. cactorum* infection.

Identification of resistance genes and an increased understanding of their expression pattern are very important to breed more resistant cultivars and/or to develop more effective disease management in the strawberry production. In the presented work, the expression of RGAs of NBS-LRR class in diploid strawberry facing *P. cactorum* infection was investigated using NBS profiling technique. The expression pattern of a limited number of RGAs was further monitored by real-time RT-PCR. This study constitutes a first step toward dissecting the molecular mechanisms of crown rot resistance in strawberry through identifying strawberry NBS-LRR RGAs to the causal agent *P. cactorum*.

## Materials and methods

### Biological materials

One isolate of *Phytophthora cactorum* (NIBIO isolate ID number 10300) was originally isolated from a diseased strawberry crown from a strawberry field at Ås, Norway. *P. cactorum* was routinely cultured on 10 % V8 agar at 25 °C (Erwin and Ribeiro 1996). Zoospore suspensions of *P. cactorum* were prepared as described previously (Chen et al. 2011).

Earlier 60 accessions/genotypes of diploid *Fragaria* sp. were tested for susceptibility to *P. cactorum* in greenhouse (Eikemo et al. 2010). Genotype FDP821 (*Fragaria vesca* subsp. *americana*) was graded as highly susceptible, while genotype Bukammen (*F. vesca*) scored as resistant. The accession FDP821 was obtained from East Malling Research, East Malling, UK and Bukammen from NIBIO, Kvithamar, Norway. These genotypes were propagated as runner plants for the experiments. Propagation was done in a greenhouse at 16 h day/20 °C and 8 h night/14 °C. High-pressure sodium lamps (SON/T, 120 μE s^−1^ m^−2^) were used to provide artificial light during the periods with less than 16 h of natural light. After multiplication and establishment, the plants were grown for an additional 1–2 weeks before inoculation with the pathogen.

### Strawberry inoculation

Strawberry plants were inoculated with *P. cactorum* isolate 10300. Each plant was gently wounded by scraping their rhizome surface with a sterile scalpel and inoculated by adding 2 ml of the zoospore suspension (1 × 10^5^ spores ml^−1^) onto the crown and lower parts of the plant with a pipette. Plants inoculated with sterile distilled water onto wounded crowns served as controls. All the treated plants were grown at 20 °C. Tissue samples of the crown area (transition zone between root and shoot) were sampled at 0, 2, 4, 6, 24, 48, 96, and 192 h post-infection (hpi). A number of replicate plants for each time point varied from 3 to 7, and the experiment was biologically repeated twice. Additional strawberry plants of each genotype, inoculated and mock-inoculated, were kept for extra days after sample harvesting. These were used to follow the disease progression and, hence, to ensure the success of the inoculations. All collected samples were immediately put into liquid nitrogen and stored at −80 °C until RNA extraction.

### Isolation of RNA and cDNA synthesis

For each set time point, total RNA was extracted from 3 to 7 inoculated strawberry crowns using Qiagen RNeasy Plant Mini Kit (Qiagen, Hilden, Germany). The quality and yield of RNA samples were measured using Agilent RNA 6000 Nano Kit on Agilent 2100 Bioanalyzer (Agilent Technologies, CA, USA).

Prior to cDNA synthesis, RNA samples were treated with DNase I (TURBO DNA-free Kit, Ambion). cDNAs were synthesized from 1-µg total RNA in a 20-µl reaction using the random primer and Superscript III Reverse Transcriptase (Invitrogen, MA, USA) according to the manufacturer’s instructions. SYBR green real-time RT-PCR (without reverse transcriptase) with primers for the house-keeping gene *actin* (Table 1) was used to check that RNA samples were not contaminated with genomic DNA. cDNAs from each RNA sample extraction were made using BD SMART PCR cDNA Synthesis Kit (Clontech, CA, USA) according to the manufacturer’s instruction and stored at −20 °C for later use.

**Table 1:** Primers used for real-time RT-PCR expression analysis of strawberry RGAs

Gene name	Accession no.	Amplicon size (bp)	Forward primer (5′–3′)	Reverse primer (5′–3′)
RGA2	JZ390871	80	TTCAGGAATTTGATGGATTGAA	AGAACCAACTCCTGAGAATGC
RGA5	JZ390872	60	TTTGGAGATCCATAGATGTTGC	GTAGCATGATGCGGCTACCA
RGA37	JZ390875	81	TGCAGAGTTGTGCAAAAGGT	GAGTGGTTGCTCTCATCATCC
RGA52	JZ390877	110	GGATGTCAACCTGTGGGGAAG	CCCCAAAACAGTATGATGCTA
RGA109	JZ390878	66	TGCTCGTCTGGTGACTTCAA	AGATCCCAACCTGATGCAAT
RGA116	JZ390879	118	CACCCATGGATTTTCTCGTT	GAACTTGTGGAGAAAATTGTGGA
RGA124	JZ390880	116	TGGGAGAAGAGGGATCAACA	ACCATGGCAAAGGATACCAG
RGA144	JZ390881	106	GCCTCCATTACTGGCTTCTTC	ACGGGAAACTGGCTCTCTTT
RGA147	JZ390882	124	GCAAAACGGAAAGATCTTGATT	GACAGGCAAACCGTCTTCTG
RGA156	JZ390883	115	GGTCCTGTCCATTGAAGAC	AAAAGGTTGCAGTGCTGGAG
RGA159	JZ390884	110	CTGGTGTTGCTCTTCATCCA	TGCAGAGCCAAGTGAAGACT
RGA169	JZ390887	74	ATATGCCAATCATGCGAACA	AATCGATTCTCGGGTAGAGGA
RGA173	JZ390888	110	CTGGAGTCCTGGCTCATGTT	TTGTTCAAGGGGATGACCTC
RGA176	JZ390889	70	GTGGACCCCAATGAGAGTGA	TACAAGACAAGCCATGGATAAGG
RGA194	JZ390891	100	TTGATTTGATTCCCCCTGAG	ATGATCAGCCACCTGCATTT
RGA195	JZ390892	94	CCAACCAGTTCGTCTTCCAT	GATCCCAAGGAGCAGCTACA
RGA196	JZ390893	135	ATGCCCCAAACAGAAACAAC	ACAACAACTGAGGCGGACTT
*Actin*	XM_011471474	106	CTTTTGGATTGAGCCTCGTC	ACGAGCTGTTTTCCCTAGCA

### NBS profiling

The samples from the resistant genotype Bukammen and the susceptible genotype FDP821 and four combinations of NBS-specific primers (NBS2, NBS3, NBS5, and NBS7) (van der Linden et al. 2004) were used in the NBS profiling. It was performed as described by van der Linden et al. (2004) with some modifications. First, NBS profiling was applied to cDNA instead of genomic DNA. This can be instrumental in identifying those members of an *R* gene cluster that are transcribed, and thus putatively functional. Second, restriction digestion and adaptor ligation were made in the same 60-μl reaction in one step. The 60-μl reaction mixture contained 200 ng of cDNA, 10 mM Tris-HAC (pH7.5), 10 mM MgAc_2_, 50 mM KAc, 5 mM DTT, 50 ng BSA, 1.2 nmol adaptor mix, 1 mM ATP, 10 U *Rsa* I, and 1 U T4 DNA ligase. The reaction was incubated at 37 °C for 3 h, and enzymes were inactivated at 65 °C for 15 min. The restriction/ligation mixture was diluted twofold with sterile water and used as template for PCR reactions.

Amplification of NBS-specific fragments was performed through a single PCR with NBS-specific primer and adaptor primer, rather than the 2-step PCR described in the original protocol (van der Linden et al. 2004). The 25-μl PCR reaction mixture consisted of 5-μl template cDNA, 20 pmol of both adaptor primer and NBS primer, 0.2 mM dNTPs, 0.2 U HotStarTaq (Qiagen), and 2.5 μl HotStarTaq PCR buffer (with 1.5 mM MgCl_2_). The PCR reaction was: 15 min at 95 °C (to activate HotStarTaq polymerase), 35 cycles at 95 °C for 30 s for denaturing, 1.4 minat 55 °C (NBS5 and NBS7) or 60 °C (NBS2 and NBS3) for annealing, 2 min at 72 °C, and a final extension at 72 °C for 20 min.

Afterward, a second similar PCR reaction was performed using 5 μl of the tenfold diluted first PCR product as template, and the same NBS-specific primer but now labeled radioactively using [γ-^33^P]dATP. The NBS-specific primer labeling was performed using T4-polynucleotide kinase from (New England Biolabs—NEB, MA, USA) as described by Sambrook et al. (1989).

The PCR products were separated by electrophoresis on 6 % polyacrylamide gels at 110 W for at least 3 h depending on the expected size of DNA fragments. After this, the gels were fixed on Whatman 3MM paper and dried at 80 °C for 1 h. BioMax MR films (Kodak, NY, USA) were exposed to the dried gels at −80 °C to visualize individual fragments.

In total, four NBS-specific primers (NBS2, NBS3, NBS5, and NBS7) and one enzyme (*Rsa*I) were used. NBS2, NBS5, NBS7, and adaptor primer were the same as described by van der Linden et al. (2004), while the NBS3 was described by Wang et al. (2008). Positions of the NBS primers in the NBS domain are referred to in these papers.

### Sequence analysis of NBS fragments

Electrophoresis and recovery of DNA fragments upregulated in the resistant genotype Bukammen and not in the susceptible genotype FDP821 were cut out from the dried polyacrylamide gels. The DNA recovery was performed as described by Liang et al. (2008), reamplified with PCR conditions identical to the first PCR of the NBS profiling protocol and purified with QIAquick PCR purification kit (Qiagen). After verification on agarose gels, PCR products were directly sequenced using the adaptor primer as a sequencing primer with the BigDye Terminator v3.1 Cycle Sequencing Kit (Applied Biosystems, MA, USA) and an ABI PrismTM 3100 Genetic Analyzer (Applied Biosystems) at the Norwegian University of Life Sciences (Ås, Norway). Sequences were searched against NCBI non-redundant database (http://www.ncbi.nlm.nih.gov) and the Genome Database for *Rosaceae* (GDR) (http://www.rosaceae.org/). The searches were performed using BLASTx (Altschul et al. 1997) with an expected (*E*) value cutoff of 10^−4^.

### SYBR green real-time RT-PCR assays

Primer pairs (Table 1) were designed using Primer Express software v2.0 (Applied Biosystems). The specificity of the amplicons was checked by melting-curve analysis and by 3 % agarose gel electrophoresis. The amplification efficiency of all primer pairs was evaluated from the slope of standard curves where efficiency = 10^−1/slope^ (Pfaffl 2001), and strawberry genomic DNA was used as a template. The strawberry housekeeping gene encoding *actin* was selected as the constitutively expressed internal control.

cDNA was diluted tenfold, and quantitative PCR was performed using 4 µl cDNA in a 25-µl final volume with Power SYBR green master mix (Applied Biosystem). The thermal cycling conditions included an initial heat-denaturing step at 95 °C for 10 min, 40 cycles of denaturing (95 °C for 15 s) and combined annealing and elongation (1 min, 60 °C). Amplification was followed by melting curve analysis and determination of melting temperature (Tm) for the PCR products, as a control of amplification specificity. All reactions were performed in technical triplicate and three non-template (water) controls were included for each primer pair. The experiment was biologically repeated twice.

The expression level was estimated by the threshold cycle (Ct) values of each gene. All results were analyzed using BestKeeper (Pfaffl et al. 2004) for calculation of the normalization factor. After normalization, the expression levels of all genes in different samples were compared against their expression in a sample selected as calibrator (see details in Results), using Ct values with the Relative Expression Software Tool (REST) (Pfaffl et al. 2002). The statistical significance was calculated by REST software using a pairwise fixed reallocation randomization test (Pfaffl et al. 2002).

### Gene cloning and sequence analysis

For cloning of the gene corresponding to RGA fragment 109, the annotated strawberry reference genome was used to design primers (primer sequences listed in Table 1, RGA 109 Forward: 5′–3′; Reverse: 5′–3′). The cDNA was used as PCR template to amplify the gene coding sequences. Cycling conditions for 30 cycles were 95 °C for 15 s, 52 °C for 30 s, and 72 °C for 2 min, with denaturation at 94 °C for 5 min and elongation at 72 °C for 5 min subsequently. The amplified products were purified and cloned into pCR2.1-TOPO vector (Invitrogen), and five clones were sequenced by Sanger-based methods. For genes larger than 1 kb in length, additional gene-specific primers for sequencing were designed at an interval of 700 base pairs.

The open reading frames (ORFs) of genes were predicted using ORF Finder (http://www.ncbi.nlm.nih.gov/gorf/gorf.html). Sequence alignments were conducted to investigate the relationship of the strawberry RGAs with other plants using the publicly available program BioEdit (Hall 1999). The full-length sequence, translated using BioEdit, was used to perform a protein–protein BLAST (BLASTP) search in NCBI database (http://www.ncbi.nlm.nih.gov/), and the protein sequences of other plant species were downloaded from the database. These sequences were then employed for multiple sequence alignments using BioEdit.

## Results

### Isolation of strawberry RGAs differentially expressed during the *P. cactorum* infection

To characterize RGAs involved in the strawberry response to *P. cactorum* infection, we identified RGAs differentially expressed upon infection using NBS profiling. For this purpose, the RGA expression profiles in a resistance genotype (Bukammen) and a susceptible one (FDP821) were investigated during an infection time course (2, 4, 6, 24, 48, 96, and 192 hpi) with *P. cactorum*. We anticipated that the RGAs present in the resistant genotype Bukammen and not in the susceptible FDP821 could be involved in strawberry plant disease resistance. Four combinations of NBS-specific primers (NBS2, NBS3, NBS5, and NBS7) with *Rsa* I were used in the NBS profiling. Each combination gave approximately 20–35 bands per lane.

### Sequence analysis

A total of 204 differentially expressed fragments were selected from the resistance genotype Bukammen for further studies (Table 2). After reamplification and purification, the PCR products were sequenced directly without cloning. From the total number of fragments, 89 gave good quality sequences, which were used for searching in the NCBI and GDR databases. Of these 89 sequences, 36 (40 %) showed significant similarity to RGA sequences, while 53 (data not shown) were similar to other genes encoding, e.g., ATP synthase b subunits, ras proteins, ribosomal elongation factors, and adenylate kinase. Of the 36 fragments showing similarity to RGAs, 13, 15, and 8 were derived from the NBS-profiling involving primers NBS2, NBS3, and NBS5, respectively, while no RGAs resulted from the NBS7 primer (Table 2). Some of the 36 RGA fragments were multiplications, which resulted in 23 unique RGA fragments (Table 3). Some fragments were only expressed in the resistant genotype before infection (RGAs 2, 5, 109, and 169), while others were expressed in the early hours after infection (RGAs 124, 168, and 180) or as late as 196 h after infection (RGAs 156 and 159). In addition, a few fragments, like RGA116, were expressed at all harvesting points after infection (Table 3). Most (21) of these RGA fragment sequences gave significant hits (*E* ≤ 10^−4^) to strawberry genes with the characteristic NBS-LRR motifs in NCBI and (or) GDR databases (Table 3).

**Table 2:** Summary of fragments derived from the *P. cactorum* resistant genotype Bukammen

Primer	Polymorphic fragments	RGA	Other genes	Low quality sequence
NBS2	56	13	12	31
NBS3	42	15	17	10
NBS5	57	8	15	34
NBS7	49	0	9	40
Total	204	36	53	115

**Table 3:** Sequence homology of 23 unique RGAs from woodland strawberry (*FragariaF. vesca*) using BLASTx searches with an expected (*E*) value cutoff of 10^−4^ in databases (NCBI and GDR)

Fragment no.	Multiplication no.	Accession no.	Length (bp)	GenBank best hit	Species best hit	*E* value	Pos.^a^ (%)	GDR best hit^b^ (gene ID)	*E* value^d^	Primer/hpi (hours post infection)^c^
RGA2		JZ390871	191	Disease resistance protein At5g66900-like	*F. vesca* subsp. *vesca*	3e−24	96	24038	1.00e−22	NBS5/0 h
RGA5		JZ390872	97	Disease resistance protein RPM1-like	*F. vesca* subsp. *vesca*	4e−12	100	27526	5.00e−12	NBS5/0 h
RGA23		JZ390873	206	Disease resistance protein At5g66900-like	*F. vesca* subsp. *vesca*	2e−28	100	08382	6.00e−27	NBS5/48 h
RGA35		JZ390874	195	Disease resistance protein RGA3-like	*F. vesca* subsp. *vesca*	2e−27	96	12852	4.00e−24	NBS5/72 h
RGA37	RGA51	JZ390875	188	Disease resistance protein RGA4-like	*F. vesca* subsp. *vesca*	9e−24	100	18750	6.00e−06	NBS5/72, 192 h
RGA44		JZ390876	184	Disease resistance protein RGA4-like	*F. vesca* subsp. *vesca*	3e−13	97	No significant hits		NBS5/96 h
RGA52		JZ390877	172	Disease resistance protein RPM1-like	*F. vesca* subsp. *vesca*	4e−11	88	07530	2.00e−11	NBS5/192 h
RGA109		JZ390878	116	TMV resistance protein N-like	*F. vesca* subsp. *vesca*	2e−06	66	034170	3.00e−06	NBS2/0 h
RGA116	RGA123, RGA155, RGA170, RGA188	JZ390879	243	Disease resistance protein At4g27190-like	*F. vesca* subsp. *vesca*	3e−34	97	24671	9.00e−20	NBS2/2, 24, 96, 192 h; NBS3/2,24 h
RGA124		JZ390880	231	Disease resistance protein RGA4-like	*F. vesca* subsp. *vesca*	8e−34	96	No significant hits		NBS2/24 h
RGA144		JZ390881	241	Disease resistance protein RGA2-like isoform 2	*F. vesca* subsp. *vesca*	2e−35	96	05851	4.00e−31	NBS2/96 h
RGA147		JZ390882	197	Disease resistance protein RGA3-like	*F. vesca* subsp. *vesca*	2e−12	74	11851	2.00e−22	NBS2/96 h
RGA156		JZ390883	234	Disease resistance protein RPH8A-like	*F. vesca* subsp. *vesca*	4e−25	87	27649	3.00e−30	NBS2/192 h
RGA159		JZ390884	200	Disease resistance protein RPM1-like	*F. vesca* subsp. *vesca*	2e−14	97	20078	9.00e−15	NBS2/192 h
RGA164		JZ390885	138	No significant hits				No significant hits		NBS3/0
RGA168	RGA171	JZ390886	155	Disease resistance protein At4g27190-like	*F. vesca* subsp. *vesca*	2e−09	80	08670	2.00e−10	NBS3/0, 2 h
RGA169		JZ390887	149	TMV resistance protein N-like	*F. vesca* subsp. *vesca*	2e−09	82	02768	3.00e−08	NBS3/0 h
RGA173	RGA193	JZ390888	284	Disease resistance protein RPM1-like	*F. vesca* subsp. *vesca*	9e−35	97	13754	8.00e−32	NBS3/4, 72 h
RGA176	RGA198	JZ390889	189	Uncharacterized protein LOC101311792	*F. vesca* subsp. *vesca*	2e−18	91	24367	5.00e−08	NBS3/4, 72 h
RGA180		JZ390890	173	Disease resistance protein At1g50180-like	*F. vesca* subsp. *vesca*	9e−13	76	12357	2.00e−13	NBS3/6 h
RGA194		JZ390891	237	Disease resistance protein At4g27190-like	*F. vesca* subsp. *vesca*	7e−30	90	08672	2.00e−31	NBS3/72 h
RGA195		JZ390892	259	No significant hits				No significant hits		NBS3/72, 96
RGA196		JZ390893	217	Disease resistance protein At1g50180-like	*F. vesca* subsp. *vesca*	1e−07	71	07226	5.00e−10	NBS3/72 h

The unique RGAs are listed in the first column, and bands resulting in identical hits and, therefore, multiplications are shown in the second column

^a^Pos, percentage of similar amino acids

^b^Database selected for BLASTx is fvesca_v1.0_genemark_abinitio.faa

^c^ *hpi* hours post-infection

^d^ *E*-value cutoff was set as 1e-4

### Expression patterns of RGAs during the *P. cactorum* infection

All of 23 RGA fragment sequences were initially considered for real-time RT-PCR analyses to confirm the appropriateness of the applied technique as well as to monitor expression profiles of the corresponding genes during strawberry infection by *P. cactorum*. Six of the designed primer pairs failed to produce a single fragment, making them inappropriate for real-time RT-PCR. Therefore, 17 genes were studied in detail using real-time RT-PCR (Table 1).

**Fig. 1:**
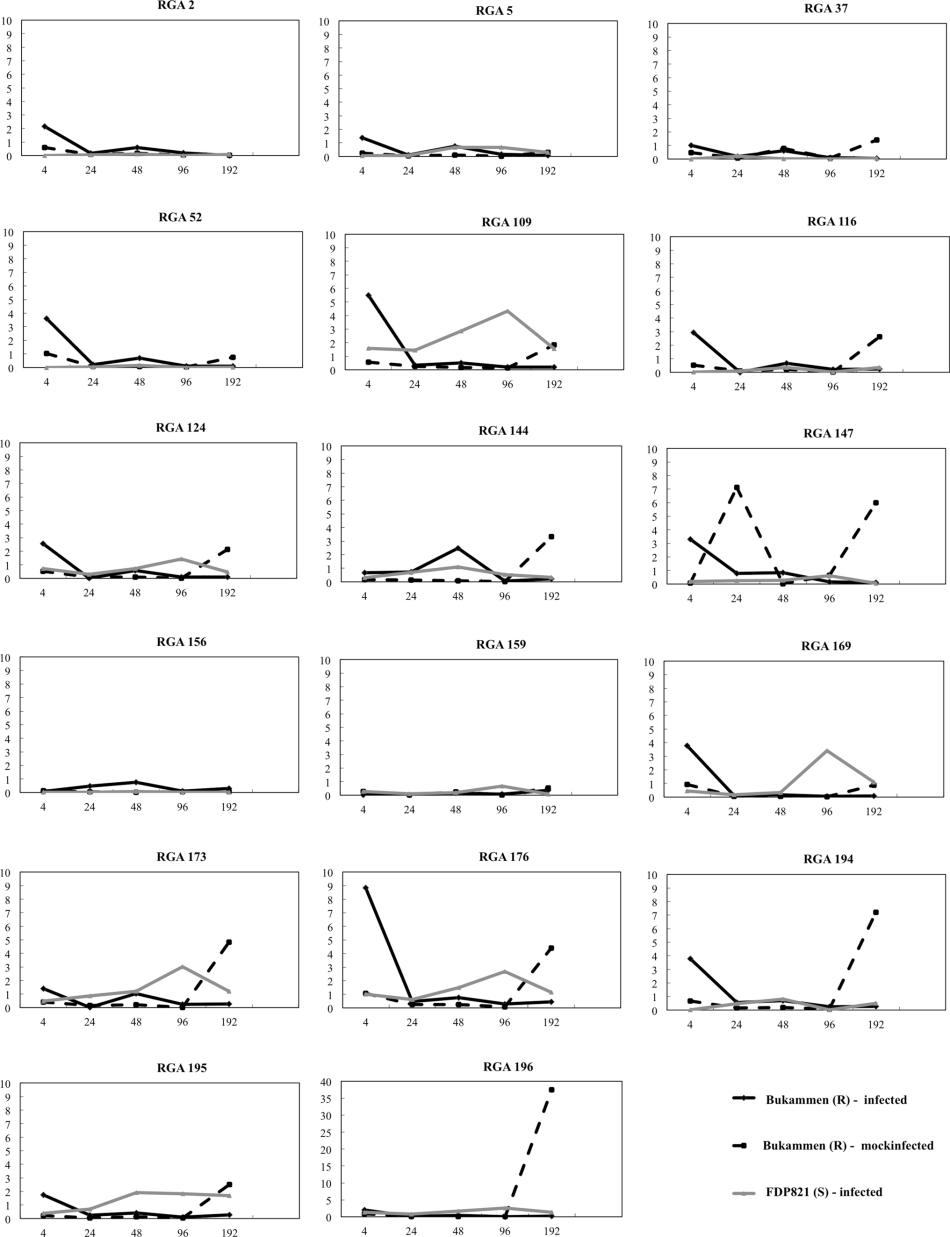
Expression profiles of 17 strawberry RGAs during a *P. cactorum* infection course [4, 24, 48, 96, and 192 h post-infection (hpi)]. Samples from the susceptible FDP821 genotype (inoculated) are labeled FDP821. Those not labeled ‘FDP’ came from the *P. cactorum* resistant Bukammen genotype in which ‘Infected’ means inoculated with *P. cactorum*, and ‘Wounded’ means only wounded by razor and mock-inoculated. Expression values for all genes are relative to those of Bukammen 0 hpi (calibrator)

Real-time RT-PCR analyses were performed using RNA isolated from the crown samples of the susceptible (FDP821) and resistance (Bukammen) genotypes interacting with the pathogen. The gene expression profiles show that the response of most of the RGAs (15 out of 17 genes) was quicker and stronger in the resistance genotype Bukammen than in the susceptible FDP821 during the very early infection stage (4 hpi) (Fig. 1). The expression of the other two genes (RGAs 159 and 196) was not significantly affected by the pathogen infection at 4 hpi. Surprisingly, the expression level of 11 RGAs (RGAs 37, 109, 116, 124, 144, 159, 173, 176, 194, 195, and 196) peaked at 192 hpi in Bukammen plants that were wounded but not inoculated. In FDP821, the expression of 8 RGAs (RGAs 2, 37, 52, 116, 147, 156, 194, and 196) was relatively stable through the infection, whereas the other 9 RGAs peaked at either 48 or 96 hpi.

One RGA (RGA109) was selected to be the first priority of cloning and further studies, because this gene was highly induced at the early infection stage (Fig. 1). As shown in Fig. 1, in Bukammen, the expression of RGA 109 peaked (5.5-fold) at 4 hpi, then dropped sharply.

### Cloning of RGA109 and sequence analysis

For full length cloning of the gene ORF corresponding to fragment No. 109, primers were designed based on the full-length ORF sequence of GDR 034170 (Table 3). PCR was performed using the cDNA sample derived from the infected Bukammen crowns at 4 hpi. After cloning and sequencing, a 1892-bp fragment was obtained, which is a bit longer than the database annotated GDR 034170 cDNA sequence (1407 bp).

**Fig. 2:**
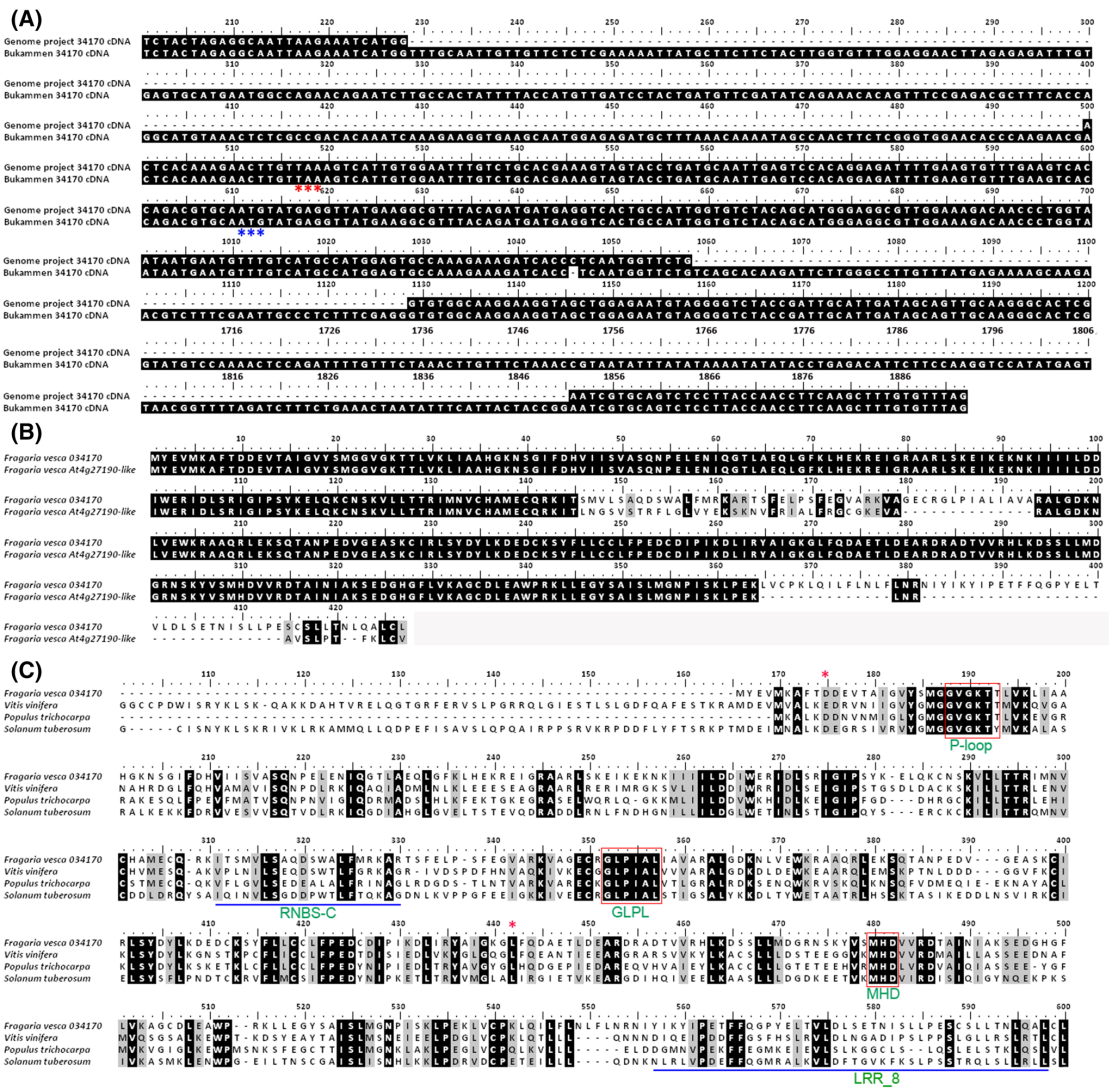
Sequence alignments. **a** Bukammen 34170 cDNA was compared with the GDR 034170 of strawberry genome database. The identical sequences were not presented unless when it was necessary to show the stop (position 517) and start (position 611) codons (marked by *three asterisks*), so that the numbers on the top of the ruler was not continuous. **b** Bukammen 34170 protein was compared with the best similarity hit, strawberry At4g27190-like protein, from NCBI. **c** Bukammen 34170 protein was aligned with the homologues from other plant species. *V. vinifera*, accession no. XP_002263288; *P. trichocarpa*, accession no. XP_002332822; *S. tuberosum*, accession no. BAD16724. The segment of sequences between *two red asterisks* (position 10-271) was characterized as NB-ARC domain, while the region from position 384–425 as LRR_8 domain, based on BLASTP search against NCBI. Motifs were annotated based on the previous publications

This gene cDNA fragment showed 96 % identity with GDR 034170 cDNA sequence but with three extra segments of sequences (positions 229–499, 1058–1127, 1706–1850) and one cytosine nucleotide deletion between position 1045 and 1046 (Fig. 2a). If the start codon was at position 1 as predicted by the automatic annotation, however, at position 517, there was a stop codon ‘TAA.’ This stop codon would lead to a non-functional protein of only 172 amino acids long. If it was the case, this indicated that in Bukammen, GDR 034170 was apparently a pseudogene. However, as shown in Fig. 1, this gene in Bukammen was highly expressed during the early infection stage. The results suggested that GDR 034170 could be mis-annotated by the automatic annotation process. This is strongly supported when another start codon at position 611 in the Bukammen cDNA sequence is detected that leads to one putative NBS-LRR protein of 427 amino acids long but without a stop codon (Fig. 2a). This means that we have not sequenced the whole gene, but only its 5′ end (position 611–1892). This putative protein sequence was used in the following analysis.

BLASTP searches show that the Bukammen 034170 protein is highly similar to a number of putative resistance proteins in *F. vesca* subsp. *vesca* and other plant species as well. The Bukammen 034170 protein gave the best similarity (89 % identity; *E* value = 0) to one disease resistance protein, At4g27190-like of strawberry line ‘Hawaii 4’. They shared two segments of amino acids (Fig. 2b). The best similarity in other plants was found to be a *Vitis vinifera* disease resistance At4g27190-like protein (54 % identity; *E* value = 9e−144) (Fig. 2c). Multiple sequence alignment showed that the Bukammen 034170 protein contains both NBS-ARC and LRR regions and the characteristic motifs, such as P-loop, RNBS-C, GLPL, MHD, and LRR, were shared between *F. vesca*, *V. vinifera*, *Populus trichocarpa,* and *Solanum tuberosum* (Fig. 2c). The results indicated that GDR 034170 in Bukammen is probably an NBS-LRR *R* gene.

## Discussion

So far, most components and mechanisms of the strawberry disease defense network remain marginally understood. Only a few published studies in strawberry have focused on gene discovery related to the mechanism of disease defense, and these have been related to defense against *Colletotrichum acutatum* (Casado-Díaz et al. 2006; Encinas-Villarejo et al. 2009; Guidarelli et al. 2011). In our study, we detect *RGA* genes differentially expressed in the resistant strawberry genotype Bukammen relative to susceptible FDP821 after infection with *P. cactorum*. Although the handling of genomic DNA as template is easier and the average percentage of polymorphic fragments obtained using genomic DNA is higher than for cDNA (Brugmans et al. 2008), using cDNA as template can be instrumental in identifying *R* genes that are transcribed, and thus putatively functional. A total of 204 cDNA-derived polymorphic fragments from Bukammen were collected, amplified, and sequenced for characterization. Out of these, 89 cDNA derived fragments gave good quality sequences, of which 36 cDNA derived fragments were similar to *R* gene or RGA sequences, resulting in 23 distinct strawberry RGAs (Table 3). Recently, 144 NBS-LRR genes were predicted from the sequenced diploid strawberry genome (Zhong et al. 2015). The reason why NBS profiling allowed us to detect a few *RGA* genes from strawberry could be first due to the sequencing strategy that we used. Cloning of fragments before sequencing could have increased the number of good quality sequences and, therefore, also the number of RGA fragments. In addition, the NBS-specific primers were originally designed on the basis of an alignment of NBS-LRR class of known *R* genes (van der Linden et al. 2004; Wang et al. 2008). Although such degenerate primers can recognize a large number of NBS-LRR gene family members, it is conceivable that the primer selectivity is biased toward already known *R* genes. With sequence data accumulating in a number of plant species, it should be possible to further optimize primer sequences to efficiently target more RGAs. Nevertheless, the results of this study showed that a relatively high number of the fragments (17.6 %) were truly derived from RGAs. This is in agreement with the previous findings of, e.g., both van der Linden et al. (2004) and Brugmans et al. (2008) who found that the majority of the fragments amplified by NBS profiling were RGA derived.

A few studies have shown that some *R* genes are upregulated in response to pathogen invasion, suggesting that plants can establish a state of increased sensitivity to pathogen attack (Navarro et al. 2004; Zipfel et al. 2004; Casado-Díaz et al. 2006). Of the 23 RGAs obtained, in our study, 6 originated from the control samples (Bukammen crowns wounded only) at time 0 hpi, while the majority of differentially expressed fragments after infection were isolated from 72, 96, and 192 hpi (Table 3; Fig. 1). This result indicated that the resistant Bukammen contains some resistance genes that are constitutively expressed, while others are induced by infection which is in line with the situation described in potato (Ronnings et al. 2003), strawberry (Casado-Díaz et al. 2006) and banana (Emediato et al. 2013). The expression profile of seventeen of the RGAs identified by NBS profiling showed that the response of most RGAs (15/17) was quicker and stronger in the resistance genotype Bukammen than in the susceptible one ‘FDP821’ during the early infection stage (Fig. 1). This is the same as described in *Musa acuminate* ssp. *burmannicoides* leaves inoculated with the fungal conidiospores of *Mycosphaerella musicola* (Emediato et al. 2013). They found that both in susceptible and resistant genotypes, some RGA genes were constitutively expressed across the infection time course, while some RGAs had low levels of expression at early infection stages and were upregulated in late stages. These results, together with our findings, highlight the importance of considering the transcriptional changes of RGAs during the pathogen infection to fully understand the mechanism of defense.

Resistance gene-mediated breeding is the most suitable strategy to control diseases and genetic transformation of *Fragaria* has made notable progress (Oosumi et al. 2006). Strawberry plants resistant to fungal diseases have been made using genes coding for chitinase from rice and Chile tomato, respectively, and a rice thaumatin II gene (*thau II*) (Asao et al. 1997; Chalavi et al. 2003; Schestibratov and Dolgov 2005). However, none of these genes belong to the NBS-LRR category of *R* genes. The exact roles of NBS-LRR RGAs obtained in strawberry defense against *P. cactorum* infection are still to be determined. Based on our findings, we postulate that the 5′ end of one RGA (GDR034170) in Bukammen has been cloned, and the characteristic motifs shared with other plant species were detected (Fig. 2). Further studies will include isolation of full-length cDNAs of selected genes, gene knockout or RNA interference to test their functions in crown rot resistance. Candidate gene application via plant transformation or marker-assisted selection will benefit strawberry breeding programs for the generation of resistant genotypes.

The results presented here clearly demonstrate that NBS profiling is a powerful technique that relatively easy isolates strawberry RGAs with altered expression in response to infection. The molecular basis of the interaction between diploid strawberry (*F. vesca*) and *P. cactorum* from a plant defense perspective has been characterized, and the results provide a first insight into the strawberry NBS-LRR RGAs responding to *P. cactorum* infection at molecular level. A comprehensive analysis of RGAs in the future will lead to a better understanding of the mechanisms involved in strawberry plant defense and contribute to the design of molecular strategies to improve disease resistance of strawberry plants.

## Abbreviations

RGA: Resistance gene analog
Hpi: Hours post-infection
NBS-LRR: Nucleotide binding site and leucine-rich repeat
